# *mcr-1* Identified in Fecal *Escherichia coli* and Avian Pathogenic *E. coli* (APEC) From Brazil

**DOI:** 10.3389/fmicb.2021.659613

**Published:** 2021-04-20

**Authors:** Nicolle Lima Barbieri, Ramon Loureiro Pimenta, Dayanne Araujo de Melo, Lisa K. Nolan, Miliane Moreira Soares de Souza, Catherine M. Logue

**Affiliations:** ^1^Department of Population Health, College of Veterinary Medicine, University of Georgia, Athens, GA, United States; ^2^Department of Veterinary Science, Universidade Federal Rural do Rio de Janeiro, Seropedica, Brazil; ^3^Department of Infectious Diseases, College of Veterinary Medicine, University of Georgia, Athens, GA, United States

**Keywords:** *mcr*, colistin, *Escherichia coli*, antimicrobial resistance, poultry *E. coli*, broiler, layer

## Abstract

Colisitin-associated resistance in bacteria of food producing animals has gained significant attention with the *mcr* gene being linked with resistance. Recently, newer variants of *mcr* have emerged with more than nine variants currently recognized. Reports of *mcr* associated resistance in *Escherichia coli* of poultry appear to be relatively limited, but its prevalence requires assessment since poultry is one of the most important and cheapest sources of the world’s protein and the emergence of resistance could limit our ability to treat disease outbreaks. Here, 107 *E. coli* isolates from production poultry were screened for the presence of *mcr 1–9*. The isolates were collected between April 2015 and June 2016 from broiler chickens and free-range layer hens in Rio de Janeiro, Brazil. All isolates were recovered from the trachea and cloaca of healthy birds and an additional two isolates were recovered from sick birds diagnosed with colibacillosis. All isolates were screened for the presence of *mcr-1* to *9* using PCR and Sanger sequencing for confirmation of positive genes. Additionally, pulse field gel electrophoresis (PFGE) analysis, avian fecal *E. coli* (APEC) virulence associated gene screening, plasmid replicon typing and antimicrobial resistance phenotype and resistance gene screening, were also carried out to further characterize these isolates. The *mcr-1* gene was detected in 62 (57.9%) isolates (61 healthy and 1 APEC) and the *mcr-5* gene was detected in 3 (2.8%) isolates; *mcr-2, mcr-3, mcr-4, mcr-6, mcr-7, mcr-8*, and *mcr-9* were not detected in any isolate. In addition, *mcr 1* and *5* positive isolates were phenotypically resistant to colistin using the agar dilution assay (> 8ug/ml). PFGE analysis found that most of the isolates screened had unique fingerprints suggesting that the emergence of colistin resistance was not the result of clonal dissemination. Plasmid replicon types *IncI2, FIB*, *and B/O* were found in 38, 36, and 34% of the *mcr* positive isolates and were the most prevalent replicon types detected; *tetA* and *tetB* (32 and 26%, respectively) were the most prevalent antimicrobial resistance genes detected and *iutA*, was the most prevalent APEC virulence associated gene, detected in 50% of the isolates. Approximately 32% of the isolates examined could be classified as APEC-like, based on the presence of 3 or more genes of APEC virulence associated path panel (*iroN, ompT, hlyF, iss, iutA*). This study has identified a high prevalence of *mcr-1* in poultry isolates in Brazil, suggesting that animal husbandry practices could result in a potential source of resistance to the human food chain in countries where application of colistin in animal health is practiced. Emergence of the *mcr* gene and associated colisitin resistance in production poultry warrants continued monitoring from the animal health and human health perspective.

## Introduction

In 2019, poultry was the most consumed meat worldwide, representing 38.6% of the world’s production (OECD, 2021). The United States is one of the largest producers and consumers of chicken meat, responsible for 19.9% of the world’s production and 17.0% of world’s consumption; Brazil, is the third largest poultry producer at 13.7%, and the fourth largest consumer at 10% (USDA, 2019).

To meet the demand for chicken, developments in production including genetic improvement of stock, nutrition, poultry health, and handling have contributed to market expansion, resulting in the exponential growth of the poultry sector (FAO, 2013).

*Escherichia coli* is a Gram negative mesophilic member of the enteric microbiota of mammals and most birds. Pathogenic strains of *E. coli* are divided into groups according to clinical symptoms and mechanisms of pathogenicity, that vary in their incubation periods and duration of the disease (Kaper et al., 2004). The production of virulence factors and the mechanisms by which these factors lead to disease, allow the classification of pathogenic *E. coli* strains into groups or pathotypes that include intestinal strains (InPEC) and extra-intestinal (ExPEC) strains. In birds, extra-intestinal disease associated with the avian pathogenic *E. coli* (APEC) pathotype has been defined (Kaper et al., 2004; Nolan et al., 2020).

APEC is the etiologic agent of colibacillosis, and the disease can present itself two forms: acute, which is characterized by septicemia and high mortality, and subacute, being characterized by hepatitis, pericarditis, airsacculitis, salpingitis, and egg yolk peritonitis in layers (Barbieri et al., 2017; Nolan et al., 2020). It is estimated that 15 to 20% of the isolates from the poultry microbiota can be considered potentially pathogenic because they harbor certain virulence factors capable of causing disease (Knöbl and Ferreira, 2009). In addition to having a high prevalence, colibacillosis causes high rates of mortality and carcass condemnation at slaughter, leading to great losses for the poultry industry in Brazil. Ferreira and colleagues (Ferreira et al., 2012), analyzed data from the Animal Products Inspection Coordination, and identified colibacillosis (19.8%) as the primary cause of bird carcass condemnations in 2010 in South Brazil (Ferreira et al., 2012).

In Brazil as elsewhere, antimicrobial resistance among *E. coli* has gained significant attention especially in light of production losses and the potential exposure of consumers to AMR strains. Poultry farms have been reported as sources of isolates harboring extended spectrum beta lactamase (ESBL) resistance (Mesa et al., 2006; Smet et al., 2008); high rates of resistance to tetracycline (Miles et al., 2006; Barbieri et al., 2013), quinolones (Bezerra et al., 2016), and trimethoprim/sulfonamides (Braga et al., 2016; Bezerra et al., 2016). In order to reduce the potential risks of AMR- associated with animal production, the use of β-lactams, sulfonamides and tetracycline for farm animal use in Brazil were banned as feed additives, and only approved for therapeutic purposes with prescription (MAPA IN-26, July 9, 2009) (MAPA, 2009).

Colistin is a broad-spectrum antimicrobial member of the polymyxin family that act on Gram negative bacteria, including many species of *Enterobacteriaceae*. The two polymyxins used therapeutically include polymyxin B and polymyxin E. Colistin was widely used as a growth promoter in Brazil until 2016 (MAPA, 2017). However, it also has important human impact because of the emergence of *Enterobacteriaceae* producing carbapenemase enzymes that has resulted in reliance on colistin human treatment (CDDEP, 2015).

Since the first report of *mcr-1* (colistin) associated resistance in *E. coli* from animals and humans in China (Liu et al., 2016). Researchers worldwide have assessed historical isolates to identify potential emergence dates for *mcr* and associated colisitin resistance and current reports have identified isolates as far back as 1980 may have harbored the gene (Shen et al., 2016). *mcr* associated resistance has been identified in a range of *Enterobacteriaceae* from humans and animals (Fernandes et al., 2016a; Haenni et al., 2016; Irrgang et al., 2016; Nordmann et al., 2016; Teo et al., 2016; Veldman et al., 2016).

For many years, resistance to colistin was not considered a problem because clinical resistance was chromosomal and restricted to hospitals. In 2015, Liu and colleagues (Liu et al., 2016) found *mcr-1* was localized to an IncI2 a plasmid identified as pHNSHP45, that demonstrated high *in vitro* transmission capacity between *E. coli* and other *Enterobacteriaceae* including *E. coli* ST131, *Klebsiella pneumoniae* ST11, and *Pseudomonas aeruginosa*.

It is believed that, regardless of selection pressure, plasmid containing *mcr-1* will likely be maintained in Enterobacteriaceae populations, facilitating ease of dissemination to the human population. The high prevalence of the *mcr-1* gene in *Escherichia coli* from meat cuts (14.9%) and birds (4.9% to 28%) suggests that the gene is widely disseminated in farm animals where colisitin is used and can be subsequently transmitted to man, because colisitin as an antimicrobial is rarely used in humans (Liu et al., 2016).

*Escherichia coli* -related virulence factors include adhesins, invasins, toxins, iron uptake systems (siderophores), which are involved in colonization, and survival in the host (Kaper et al., 2004). The use of molecular techniques for detecting these genes has allowed the characterization of bacterial virulence (Johnson et al., 2008; Barbieri et al., 2015).

Johnson and colleagues (Johnson et al., 2008), studied the prevalence of 46 virulence genes in APEC and avian fecal *E. coli* (AFEC) (fecal commensal avian) strains, and found that the siderophore Salmochelin receptor virulence gene (*iroN*), a gene encoding in the episomal external outer membrane protein (*ompT*), gene encoding hemolysin (*hlyF*), the increased serum survival gene (*iss*), and aerobactin siderophore receptor gene (*iutA*) had a significantly greater prevalence in APEC compared to AFEC strains. Most APEC harbored three or more of these genes, demonstrating their presence can be used to identify potentially pathogenic strains for birds (Johnson et al., 2008). Other virulence factors of APEC include acquisition of iron through siderophores and other means that appear to play an important role in the pathogenicity of strains, especially in septicemia associated organisms allowing APEC to survive in serum where the iron concentration is extremely low (Janssen et al., 2001; Caza et al., 2008) directly influencing their pathogenesis (Gao et al., 2012). The genetic determinants involved in the pathogenicity of the APEC strains are, however, not yet fully understood (Maluta et al., 2016).

The overall goal of this study was to assess *E. coli* isolates recovered from the feces and trachea of healthy broilers in Brazil for the presence of colistin-associated resistance by *mcr* and other resistance determinants and to characterize all isolates for virulence and associated resistance traits. In addition, PFGE was performed to determine any potential genetic relatedness though DNA fingerprint analysis.

## Materials and Methods

### Isolate Collection

The analysis consisted of one hundred and seven *E. coli* isolates collected between April 2015 and March 2016 from two broiler and one layer farms located in the Rio de Janeiro area of Brazil. All isolates were recovered from the trachea and cloaca of healthy birds. An additional two isolates were recovered from sick birds (Blepharitis B46; celoma cavity B157). All isolates were recovered from 30 to 41-day-old broiler chickens and free-range layer hens at 62 weeks-of age as detailed in Supplementary Table 1.

A total of 120 swab samples, 60 from cloaca and 60 from trachea, were collected on two broiler chicken farms (1 and 2) and 30 samples from the laying hens at farm 3.

Swab samples were collected from the cloaca or trachea of healthy birds and placed in Stuart media (Absorve^®^ Jiangsu, China) for transportation. At the lab, all swabs were plated on MacConkey (MAC, HiMedia^®^, Mumbai, India) agar and Eosin Methylene Blue agar (EMB, HiMedia^®^) with incubation at 37°C for 18–24h. All suspect colonies (1 colony per sample) were confirmed as *E. coli* using MALDI-TOF MS (LT Microflex Bruker, Bruker, Germany). *E. coli* positive strains for MALDI-TOF were confirmed using a polymerase chain reaction (PCR) targeting the 16S DNA as described previously by Lamprecht et al. (Lamprecht et al., 2014). All strains were stored at –80°C in Luria-Bertani (LB) (BD Difco^TM^, Sparks, United States) broth with 20% glycerol until use.

### *mcr* PCR Analysis

All isolates were screened for the presence of the *mcr-1* to 9 gene using protocols recently described elsewhere (Supplementary Table 2) (Liu et al., 2016; Xavier et al., 2016; Borowiak et al., 2017; Carattoli et al., 2017; Yin et al., 2017; Kieffer et al., 2019; Yang et al., 2019). DNA was extracted from all strains using the boil prep method and PCR reaction preparation as described previously (Barbieri et al., 2013). All PCR amplifications were carried out under the following conditions 94°C for 10 min followed by 30 cycles of 94°C for 30 sec; 58°C for 30 sec and 72°C for 2 min; with a final extension of 72°C for 10 min.

Polymerase chain reaction products generated were subjected to electrophoresis in 2% (w/v) agarose gels (LE Agarose, Lonza, GA, United States) in 1X TAE buffer and run at 120V for 2 h. A Hi-Lo molecular weight marker (100 bp; Minnesota Molecular, MN) was used as the size standard; we used a laboratory strain (7-49-1) as a positive control for *mcr-1* (Barbieri et al., 2017) and DNAse/RNAse free water was used as the negative control. Gels were stained in 0.25% ethidium bromide (Fisher Scientific, Asheville, NC), and bands corresponding to each gene present were recorded using a UV Imager (Omega Fluor, Aplegen, Pleasanton, CA).

Sixty-two PCR products positive for the gene *mcr-1* and 3 PCR products positive for *mcr-5* were selected for sequencing. The full gene PCR product was treated with ExoSAP-IT^®^ (Affymetrix, Santa Clara, CA) to remove primer and remaining DNTPs following manufacturer’s protocols and submitted to Iowa State University’s DNA facility for Sanger sequencing of the forward and reverse strands. Sequences generated were imported into Geneious^®^ software and aligned to compare across the isolates positive for the fragment.

### Antimicrobial Resistance Analysis

The antimicrobial susceptibility of all *E. coli* isolates was examined using the disk diffusion method according to the Clinical and Laboratory Standards Institute (CLSI) guidelines (CLSI, 2017), using *Escherichia coli* strain ATCC 25922 as a control. The 8 antimicrobial agents tested included: amoxicillin (AMO; 25 μg), ceftazidine (CAZ; 30 μg), cefoxitin (CFO; 30 μg), cefotaxime (CTX; 30 μg), aztronam (ATM; 30 μg), imipenem (IPM; 10 μg), cefepime (CPM; 30 μg), and a combination of amoxicillin and clavulanic acid (AMC; 20 + 10 μg).

The breakpoints used were obtained from CLSI (CLSI, 2017) for all antimicrobials (Supplementary Table 3A).

### Colistin Antimicrobial Susceptibility Analysis

To assess the role of colistin resistance in strains positive for the *mcr* gene all strains were subjected to antimicrobial susceptibility analysis to colistin sulfate (Alfa Aesar, Ward Hill, MA) using the agar dilution assay. Overnight cultures of each strain were grown on Tryptone Soya Agar (TSA) plates and colonies selected were adjusted to an OD 0.5 Mc Farland in sterile water using an nephelometer (Sensititre); then 10 μl of the suspension was added to 11 μl of Mueller Hinton (MH) broth and mixed well using a vortex. 10 μl of this suspension was used to spot inoculate the agar dilution plates (Turlej-Rogacka et al., 2018).

The agar dilution plates tested antimicrobial resistance to colistin in doubling dilutions at the following dilution range 0.5 to 32 μg/ml. Once all plates were inoculated as appropriate, they were allowed to dry and incubated at 37°C for 18h. Plates were observed for growth and minimum inhibitory concentrations (MIC’s) were defined as the lowest concentration of antimicrobial to inhibit growth of the test strains.

### Antimicrobial Associated Resistance Genes Screening

All isolates were tested for the presence of the antimicrobial associated resistance genes: *silP; intI1; pcoD; sulI; ISEc12; aad; aac3-VI; qacE*Δ*1; blaTEM; aac3-VI; tetB; tetA; groEL; aph*(3)*IA; dfr17* (Zhao et al., 2001; Brinas et al., 2002; Maynard et al., 2004; Grobner et al., 2009) using multiplex PCR and primers described in previous studies of our lab (Supplementary Table 2).

### Plasmid Replicon Detection

Plasmids potentially associated with virulence and/or resistance in these test isolates were assessed using the plasmid replicon typing protocols as described by Carattoli et al. and Johnson and Nolan (Carattoli et al., 2005; Johnson and Nolan, 2009); in addition, *IncI2* (Zhao and Zong, 2016) using standard multiplex PCR protocols and primers as described previously (see Supplementary Table 2).

### Genotyping Avian *E. coli* for *iroN, ompT, hlyF, iss*, and *iutA*

*Escherichia coli* strains were genotyped by multiplex PCR as previously described (Johnson et al., 2008). Reactions were performed as follows: denaturation for 2 minutes at 94°C; 25 cycles of 30 s at 94°C, 30 s at 63°C and 3 min at 68°C, followed by a final extension step of 10 min at 72°C. PCR products were run on a 2% agarose gel as described above. APEC O1 strain was used as the positive control and sterile water in place of DNA for the negative control.

### Phylogenetic Typing

Samples of the DNA stock from each strain were also subjected to phylogenetic typing using the revised protocols described by Clermont et al. (Clermont et al., 2013). Here, a 25 μl PCR reaction volume as described above with the following PCR conditions: denaturation for 4 minutes at 94°C followed by 30 cycles of 5s at 94°C; 30s at 64°C (group E), or 63°C (quadruplex) or 66°C (group C) and 30s at 72°C with a final extension at 72°C for 5 min. Polymerase chain reaction products were run on a 1.5% agarose gel as described above.

### Pulsed Field Gel Electrophoresis Analysis

All strains were subjected to molecular subtyping using PFGE. Isolates were analyzed using the method of Ribot et al. and Hussein et al. (Ribot et al., 2006; Hussein et al., 2013). Preparation, lysis, washing of plugs, and *Xba*I restriction were performed according to the PulseNet protocol. *Salmonella Braenderup* H9812 was used as the size standard. Macrorestriction patterns were compared using the BioNumerics Fingerprinting software (Ver 6.6, Applied Math, Austin, TX). The similarity index was calculated using the Dice coefficient, with a band position tolerance of 1% and an optimization of 0.5%.

### Statistical Analysis

Data was analyzed using non-parametric tests due to asymmetry in the distribution of genes or other traits used for analysis.

For analysis of the association between the presence of two single genes or antimicrobial resistance traits (Supplementary Table 4A) were tested by use of the chi-square test.

For the analysis of virulence and resistance genes harbored by strains examined in the study the number of genes were treated as quantitative variables and the data was analyzed using non-parametric tests also due to asymmetry in the distribution of these genes. Direct comparisons (where possible) between two groups (Supplementary Tables 4B,C) were made using the Mann-Whitney *U* test.

All statistical analysis was performed using GraphPad Prism (Version 7.0d) for MAC OS X (GraphPad, La Jolla, CA) or IBM SPSS Statistics (Version 26.0) for MAC OS X (IBM Corp., Armonk, NY). Statistical significance was accepted when *p* < 0.05.

## Results

### Isolate Distribution

A total of 175 suspect *E. coli* strains were isolated from farm 1, 2, and 3; 80/175 from farm 1 (45.7%), 83/175 (47.4%) from farm 2, and 12/175 (6.5%) from farm 3.

**FIGURE 1:**
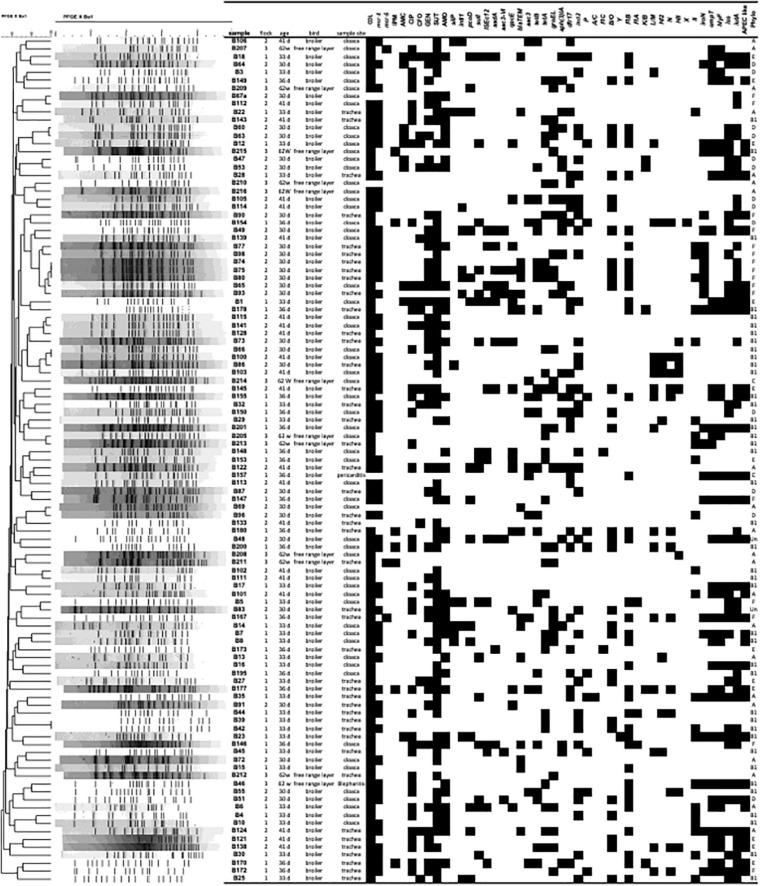
Pulsed-field gel electrophoresis (PFGE) profile of 107 avian *Escherichia coli* isolates. The PFGE dendrogram was constructed by the unweighted-pair group method with arithmetic averages. The scale indicates levels of similarity within this set of isolates based upon *Xba*I enzyme restriction digestion of total bacterial DNA. The column Sample shows isolate designation; the column flock, indicates the farm the isolate came from **(1–3)**; the column Age, days or weeks old of the bird; bird shows type of bird; Sample Site, site of bacterial isolation; the subsequent columns depict the antimicrobial resistance and PCR results for virulence genes (VAGs) tested, with presence indicated in black and absence indicated in white; Phylo, phylogenetic group.

*Escherichia coli* isolates were confirmed using the MALDI TOF MS technique for 107 isolates, with scores of 2.0 to 2.495. *E. coli* positive strains for MALDI-TOF were confirmed using a PCR targeting the 16S DNA. A total of 107 *E. coli* isolates were used for the study. Based on distribution profile by collection site, it was observed that in farm 1, a total of 26/107 (24.3%) of the isolates came from the cloaca, while 20/107 (18.7%) were recovered from the trachea. On farm 2, 27/107 (25.3%) of the isolates were isolated from the cloaca and 21/107 (19.6%) came from the trachea. On farm 3, 8/107 (7.5%) of the isolates came from the cloaca while 3/107 (2.8%) were recovered from the trachea. Two additional *E. coli* isolates were included in this analysis recovered from sick birds diagnosed with colibacillosis on necropsy (Blepharitis B46 farm 1; celoma cavity B157 farm 3) (Figure 1 and Supplementary Table 1).

### Antimicrobial Resistance Analysis

**FIGURE 2:**
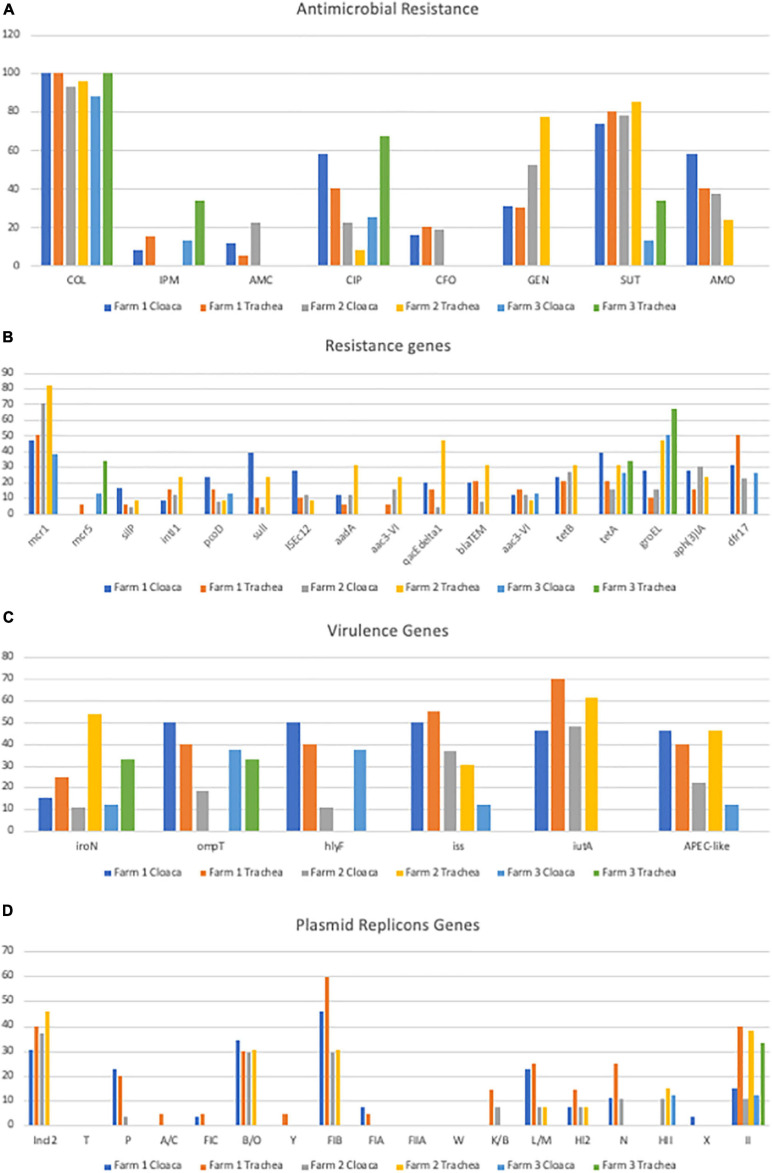
Gene prevalence separated by organ and farm **(A)** Prevalence of Antimicrobial Resistance; **(B)** Prevalence of Genes Encoding Antimicrobial Resistance; **(C)** Prevalence of Genes Encoding Virulence Factors; **(D)** Prevalence of Plasmid Replicon Genes.

Data from resistance analysis based on the disk diffusion assay are shown in Figure 1 and Table 1. Data are presented based on source of origin (cloaca vs. trachea) and farm (Figure 2A).

**TABLE 1:** Prevalence of genes tested in all samples and in *mcr* positive samples.

		All samples		*mcr* positive	
		*n*	%	*n*	%
**Antimicrobial Resistance**					
Colistin	COL	102	95.33	62	100.00
Imipenem	IPM	7	6.54	3	4.84
Amoxicillin	AMC	10	9.35	4	6.45
Ciprofloxacin	CIP	41	38.32	22	35.48
Cefoxitin	CFO	16	14.95	7	11.29
Gentamicin	GEN	45	42.06	28	45.16
Sulphonamides and trimethoprim	SUT	76	71.03	45	72.58
Amoxicillin and clavulanic acid	AMO	40	37.38	23	37.10
**Antimicrobial Associated Resistance Genes**					
Colistin resistance	*mcr1*	62	57.94	62	100.00
Silver resistance	*silP*	7	6.54	6	9.68
Integrase	*intI1*	11	10.28	8	12.90
Copper resistance	*pcoD*	15	14.02	10	16.13
Sulfa resistance	*sulI*	17	15.89	9	14.52
Transposase	*ISEc12*	14	13.08	6	9.68
Aminoglycoside resistance	*aadA*	12	11.21	7	11.29
Gentamicin resistance	*aac3-VI*	8	7.48	6	9.68
Quarternary amonium resistance	*qac delta1*	17	15.89	12	19.35
Ampicillin resistance	*blaTEM*	16	14.95	9	14.52
Gentamicin resistance	*aac3-VI*	13	12.15	9	14.52
Tetracycline resistance	*tetB*	24	22.43	16	25.81
Tetracycline resistance	*tetA*	30	28.04	20	32.26
Chaperone	*groEL*	30	28.04	21	33.87
Gentamicin resistance	*aph*(3)*IA*	22	20.56	12	19.35
Trimethoprim resistance	*dfr17*	29	27.10	16	25.81
**APEC Minimal Predictors**					
Salmochelin siderophore receptor gene	*iroN*	24	22.43	15	24.19
Episomal outer membrane protease gene	*ompT*	36	33.64	19	30.65
Putative avian hemolysin F	*hlyF*	33	30.84	17	27.42
Episomal increased serum survival gene	*iss*	42	39.25	23	37.10
Aerobactin siderophore receptor gene	*iutA*	53	49.53	31	50.00
3 or more predictors		33	30.84	20	32.26
***Plasmid Replicon Genes***					
Plasmid replicon typing	*incI2*	34	31.78	23	37.10
Plasmid replicon typing	*T*	0	0.00	0	0.00
Plasmid replicon typing	*P*	11	10.28	4	6.45
Plasmid replicon typing	*A/C*	1	0.93	0	0.00
Plasmid replicon typing	*FIC*	2	1.87	0	0.00
Plasmid replicon typing	*B/O*	30	28.04	21	33.87
Plasmid replicon typing	*Y*	1	0.93	0	0.00
Plasmid replicon typing	*FIB*	39	36.45	22	35.48
Plasmid replicon typing	*FIA*	3	2.80	1	1.61
Plasmid replicon typing	*FIIA*	0	0.00	0	0.00
Plasmid replicon typing	*W*	0	0.00	0	0.00
Plasmid replicon typing	*K/B*	5	4.67	2	3.23
Plasmid replicon typing	*L/M*	16	14.95	10	16.13
Plasmid replicon typing	*Hl2*	8	7.48	6	9.68
Plasmid replicon typing	*N*	13	12.15	9	14.52
Plasmid replicon typing	*HII*	8	7.48	7	11.29
Plasmid replicon typing	*X*	1	0.93	0	0.00
Plasmid replicon typing	*II*	24	22.43	14	22.58
**Phylogenetic Typing**					
Phylotype group	A	21	19.62	12	19.35
Phylotype group	B1	41	38.32	22	35.48
Phylotype group	B2	0	0.00	0	0.00
Phylotype group	C	2	1.87	1	1.61
Phylotype group	D	13	12.15	9	14.52
Phylotype group	E	12	11.21	6	9.68
Phylotype group	F	16	14.95	10	16.13

On farm 1, 100% (26/26) of the *E. coli* strains recovered from the cloaca showed phenotypic resistance to colistin (COL), 73% (19/26) to Sulfa-trimethoprim (SUT), 57% (15/26) to ciprofloxacin (CIP), 57% (15/26) to AMO, 30% (8/26) to gentamicin (GEN), 15% (4/26) to CFO, 11% (3/26) to AMC and 7% (2/26) to imipenem (IMP). Of the 21 strains of *E. coli* from trachea 95% (20/21) showed phenotypic resistance to COL, 76% (16/21) to SUT, 38% (8/21) to AMO, 28% (6/21) to CIP, 28% (6/21) to GEN, 19% (4/21) to CFO, 14% (3/21) to IMP and 4% (1/21) to AMC (Figure 2A).

On farm 2, 92% (25/27) of *E. coli* strains from cloaca were resistant to COL, 77% (21/27) to SUT, 51% (14/27) to GEN, 37% (10/27) to AMO, 22% (6/27) to CIP, 22% (6/27) to AMC and 18% (5/27) to CFO. Of the 21 strains originating from trachea 95% (20/21) showed resistance to COL, 85% (18/21) to SUT, 80% (17/21) to GEN, 33% (7/21) CIP, 28% (6/21) AMO and 14% (3/21) CFO (Figure 2A).

On farm 3, 87% (7/8) of the *E. coli* from the cloaca were resistant to COL, 25% (2/8) to CIP, 12% (2/8) to SUT, while in strains from trachea 100% (4/4) showed phenotypic resistance to COL, 75% (3/4) to CIP, 25% (1/4) to IMP, 25% (1/4) to SUT (Figure 2A).

Phenotype analysis of multidrug resistance found 18.7% (Nordmann et al., 2016) of the isolates were resistant to five antimicrobial agents or more; 19.6% (Haenni et al., 2016) were resistant to 4; 27.1% (Caza et al., 2008) were resistant to 3; 17.8% (Shen et al., 2016) were resistant to 2 and 14.0% (MAPA, 2009) were resistant to 1 and only 2.8% (FAO, 2013) isolates were susceptible to all agents tested (Table 2, and Supplementary Table 3). Of note, however, 3 strains was found to be resistant to 7 different antimicrobials (Table 2). The most frequent profile of resistance were COL, present in 15 isolates; COL, SUT present in 11 isolates and COL, SUT, GEN present in 12 isolates. No isolates were resistant to all eight antimicrobials tested (Table 2, Supplementary Table 3).

**TABLE 2:** Antimicrobial resistance profiles among isolates examined.

Profile	Number of Strains
COL, SUT, GEN, CIP, AMO, CFO, AMC	3
COL, SUT, GEN, CIP, AMO, CFO	3
COL, SUT, GEN, CIP, AMO, IPM	2
COL, SUT, GEN, CIP, AMO	5
COL, SUT, GEN, CIP, CFO	1
COL, SUT, GEN, CIP, IPM	1
COL, SUT, GEN, CIP	4
COL, SUT, GEN, AMO, CFO, AMC	1
COL, SUT, GEN, AMO	1
COL, SUT, GEN, AMO	3
COL, SUT, GEN, CFO	3
COL, SUT, GEN, AMC	1
COL, SUT, GEN	12
COL, SUT, CIP, AMO, CFO, AMC	1
COL, SUT, CIP, AMO, CFO	1
COL, SUT, CIP, AMO, AMC	1
COL, SUT, CIP, AMO	6
COL, SUT, CIP, IPM	1
COL, SUT, CIP	6
COL, SUT, AMO, IPM	1
COL, SUT, AMO	5
COL, SUT, CFO	1
COL, SUT	11
COL, GEN, CIP	2
COL, GEN, AMO, CFO, AMC	1
COL, GEN, AMO, AMC	1
COL, CIP, AMO	1
COL, CIP	3
COL, AMO, AMC	1
COL, AMO	2
COL, IPM	2
COL	15
SUT, GEN, AMO	1
SUT, CFO	1
Susceptible	3

*amoxicillin (AMO), ceftazidine (CAZ), cefoxitin (CFO), efotaxime (CTX), aztronam (ATM), imipenem (IPM), cefepime (CPM), and a combination ofamoxicillin and clavulanic acid (AMC).*

Among the strains of *E. coli* that displayed resistance to AMC, AMO, and CFO in the disk diffusion test, 52/107 (48.59%) were assessed for β-lactamase activity. 12/52 (23%) strains that presented phenotypic profiles compatible with those of ESBL producers, representing 11% of the total evaluated strains (12/107). In addition, two strains showed an AmpC production profile (2/52; 3%) and five (5/52; 9%) showed results compatible with the coproduction of both ESBL and AmpC enzymes, representing 2% (2/107) and 4% (5/107), of all strains examined. When the prevalence per farm was assessed, 27% (13/47) of the *E. coli* strains from farm 1 showed beta-lactamase production, 61% (8/13) ESBL, 15% (2/13) AmpC, and 23% (3/13) co-production of both enzymes, while on farm 2 10% (5/48) showed beta-lactamase production, with 60% (3/5) ESBL only and 40% (2/5) displaying enzyme coproduction. On farm 3, 8% (1/12) displayed an ESBL production profile only.

Using the Mann-Whitney *U* test, to compare antimicrobial resistance prevalence with farm of isolation we found some significant relationships (*p* < 0.05) between certain groups, including antimicrobials such as GEN and SUT in cloaca vs. trachea; IPM, GEN, SUT, and AMO in broiler vs. free range; IPM, CIP, and GEN in farm 1 vs. farm 2; GEN, SUT, and AMO in farm 1 vs. farm 3; IMP, GEN, and SUT in farm 2 vs. farm 3 (Supplementary Table 4B). Similarly, using the chi-square test (Supplementary Table 3A), that allows comparison between the presence of two antimicrobial resistances analyzed in all strains, significant associations were observed for some specific antimicrobials such as AMO and a number of other antimicrobials including AMC, CIP, CFO, and SUT (*p* < 0.05).

### Colistin Antimicrobial Susceptibility Analysis and *mcr* Analysis

Among the 107 *E. coli* strains evaluated, 102 (95.33%) were resistant to colistin using the agar dilution assay (> 8ug/ml). We found that the *mcr-1* gene was detected in 62 (57.94%) isolates (61 healthy and 1 APEC); and the *mcr-5* gene was detected in 3 (2.8%) isolates; *mcr -2, 3, 4, 6, 7, 8*, and *9* were not detected in any isolate. However, 35% (37/102) displayed phenotypic resistance to colisitin without genotype confirmation of the presence of *mcr-1* (Figure 1 and Table 1).

Using the Mann-Whitney *U* test, to compare *mcr* prevalence with farm of isolation we found significant relationships (*p* < 0.05) between certain groups, including *mcr-1* detection on farm 1 vs farm 2 and farm 2 vs. farm 3 (Supplementary Table 4B).

**FIGURE 3:**
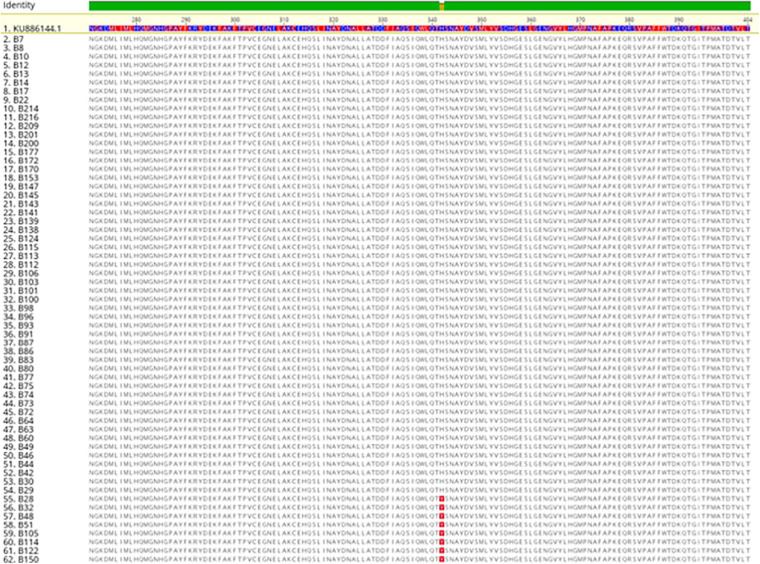
Protein Alignment of *mcr-1* positive strains examined in this study.

**FIGURE 4:**
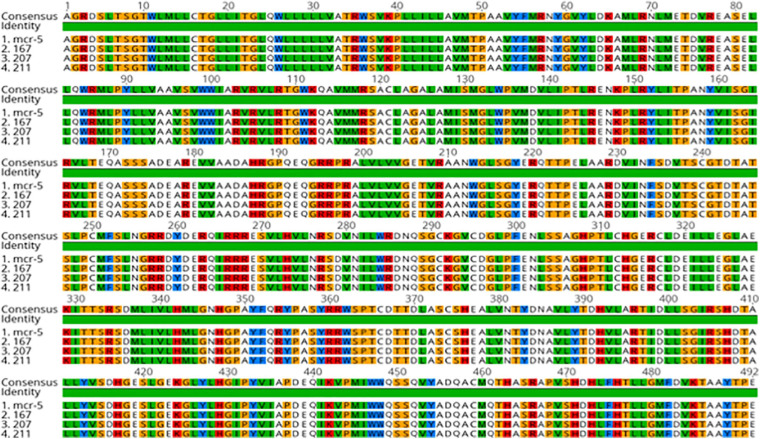
Protein Alignment of *mcr-5* positive strains examined in this study.

The sequence analysis of the 62 isolates harboring *mcr-1* (61 healthy and 1 APEC) found that 54 isolates had the exact same sequence compared with *mcr-1* in GenBank (KU886144.1) and 8 isolates have an amino acid change (H452Y) at position 452 (NG_052663.1) (Figure 3). The sequence analysis of the 3 isolates with *mcr- 5* have the identical sequence to *mcr-5* in GenBank (NG055658.1) (Figure 4).

### Antimicrobial Associated Resistance Genes Screening

Among the 107 *E. coli* strains evaluated, 94 (88%) harbored some antimicrobial resistance-associated gene. One antimicrobial resistance gene was detected in 18% (19/107) of the *E. coli* examined, two genes were detected in 17% (18/107); three genes in 16% (17/107); four genes in 15% (16/107); five genes in 7% (8/107); six genes in 6% (6/107); seven genes in 5% (5/107); eight genes in 2% (2/107); nine genes in 2% (2/107) and ten antimicrobial resistance genes in 0.9% (1/107) of strains examined (Figure 1 and Table 1).

Regarding resistance to the aminoglycoside gentamicin, there was a correlation between phenotypic and genotypic resistance in 40% (18/45) of the strains evaluated. The *aadA* gene was significantly (*p* < 0.05) associated with the phenotypic resistance observed with 75% (9/12) of the strains that had the gene, expressed resistance in the disk diffusion assay, but resistance was not statistically associated with the *aac3-VIb* genes with 50% (4/8) positive, *aph*AI (FAO, 2013) with 50% (11/22) positive and *aac3-VIa* with 38% (5/13) positive. It was also noted that some isolates harbored more than one gene associated with gentamicin (aminoglycoside) resistance (Figure 2B and Supplementary Table 4A).

Sulfa-trimethoprim was the second antimicrobial with the highest prevalence of phenotypic resistance in the disk diffusion assay (71%; 76/107), with phenotypic and genotypic correlation in 15.78% (12/76) of the strains evaluated. 12/17 (71%) strains that had the *sul1* gene and 20/28 (71%) of the strains that had the *dfr-17* gene showed phenotypic resistance to SUT. However, 64% (49/76) showed phenotypic resistance and but did not harbor either of the two genes (Figure 1 and Table 1).

The *blaTEM* gene was detected in 15% (16/107) of *E. coli* strains examined, however, only 25% (4/16) of strains that had the gene were considered to produce ESBL in phenotypic tests, this can be explained because there are extended spectrum variants of the gene that were not assayed by the PCR assays used in our analysis. The prevalence of resistance-related genes is shown in Table 1.

The gene that encodes the integrase enzyme (*intl1*) was detected in 10% (11/107) of *E. coli* strains and 13% (8/62) of *mcr-1* positive strains. The presence of the gene encoding the transposase enzyme (*ISEc12*) was also detected in 13% (14/107) of *E. coli* strains and 10% (6/62) of the positive *mcr-1* strains. *tetA* and *tetB* (32 and 25%) were the most prevalent antimicrobial resistance genes in *mcr-1* positive isolates.

Using the Mann-Whitney *U* test, to compare antimicrobial resistance prevalence with farm of isolation we found some significant relationships (*p* < 0.05) between certain groups, including antimicrobial resistance genes such as *dfr17* on farm 1 vs. farm 2; (Supplementary Table 4B). Similarly, using the chi-square test (Supplementary Table 4A), that allows comparison between the presence of two antimicrobial resistances analyzed in all strains, significant associations were observed for some specific antimicrobial resistance genes such as *qac*Δ*1* and several other antimicrobial resistance genes including *sulI*, *ISEc12*, and *aadA* (*p* < 0.05).

### Plasmid Replicon Detection

Among the seventeen plasmid incompatibility groups tested, the three most prevalent included *IncI2*, *B/O*, and *FIB* with 32% (34/107), 28% (30/107), and 36% (39/107) prevalence, respectively. (Table 1 and Figure 2D). When the prevalence of these plasmids was correlated with the presence of the *mcr-1* gene, a greater occurrence of this resistance gene was observed in the strains where these plasmids were detected separately and concomitantly (Supplementary Table 4A). Significant associations were observed for the detection of *mcr-1* and the *HII* plasmid replicon (*p* < 0.05).

*IncI2, FIB*, and *B/O* (37, 35, and 34%) were the most prevalent replicon types detected in the *mcr-1* positive isolates. We could not confirm that the *mcr-1* gene was contained on the plasmid, integron or transposon as this analysis was beyond the scope of the current study, however, the occurrence of these elements in positive *mcr-1* strains can be considered a risk factor, since the *mcr-1* gene can move to these mobile genetic elements, facilitating the dispersion of the gene. Such characteristics were observed in the B48 strain, where the genes *mcr-1*, *intl1*, *ISEc 12*, and plasmids *IncI2*, and *FIB* were detected simultaneously.

Using the Mann-Whitney *U* test, to compare plasmid replicon prevalence with farm of isolation we found some significant relationships (*p* < 0.05) between certain groups, including plasmid replicon such as *II* in the cloaca vs. trachea; *IncI2*, *FIB*, and *B/O* in broiler vs. free range birds; *P*, *FIB*, and *HII* on farm 1 vs. farm 2; *IncI2* and *FIB* on farm 1 vs. farm 3; *IncI2* on farm 2 vs. farm 3 (Supplementary Table 4B). Similarly, using the chi-square test (Supplementary Table 4A), that allows comparison between the presence of two plasmid replicons analyzed in all strains, significant associations were observed for some plasmid replicons such as *HII* and a few other plasmid replicons including *P*, *HI2* and *L/M* (*p* < 0.05).

### Genotyping Avian *E. coli* for *iroN, ompT, hlyF, iss*, and *iutA*

Within the group of virulence genes used to characterize strains such as APEC, the gene with the highest prevalence was *iutA*, followed by *iss*, *ompT*, *hlyF*, *iroN* with 49, 39, 33, 30, and 22%, respectively (Table 1 and Figure 2C). Of the 107 strains of *E. coli* analyzed, 31% (33/107) were characterized as APEC-like, as they harbored three or more virulence genes of the path panel. These strains, however, were not isolated from lesions of diseased birds but rather healthy birds.

The highest prevalence of strains characterized as APEC-like occurred on farm 1 (40%; 19/47), 38% (10/26) of cloaca and 42% (9/21) of trachea, on farm 2, 25% (12/48) of the strains were considered APEC-like, 22% (6/27) of the cloacal strains and 28% (6/21) of the trachea (Figure 2C). On farm 3, 16% (2/12) of the strains were considered potentially pathogenic, with a greater proportion detected in the trachea (25%; 1/4) compared with the cloaca (12%; 1/8). *iutA*, was the most prevalent APEC virulence associated gene in *mcr-1* positive isolates, present in 50% of the isolates and just considering *mcr-1* positive isolates, 32% could be classified APEC-like (Figure 2C).

### Phylogenetic Typing

Most strains were classified as belonging to phylogenetic group B1 (38%; 41/107), followed by group A (20%; 21/107), group F (15%; 16/107), group D (12%; 13/107), group E (11%; 12/107) and group C (2%; 2/107). None of the isolates were identified as phylogenetic group B2 (Table 1).

Thirty-four strains were considered APEC-like, with 9 (26%) and 7 (20%) distributed in phylogenetic groups B1 and F, respectively. The *mcr-1* gene was detected in 62 strains, 22 (35%) belonging to phylogenetic group B1 and 10 (16%) belonging to phylogenetic group F.

The prevalence of other phylogenetic groups and the correlation between the characterization as APEC-like and the presence of the *mcr-1* gene can be seen in Table 1 and Supplementary Table 4D.

### Pulsed Field Gel Electrophoresis Analysis

When evaluating the results obtained by Pulsed-field Gel analysis, there was great genetic diversity within the strains of *E. coli* examined. However, there was 100% identify between isolates B74, B75, B80, between B66 and B100 and between B39 and B42 (Figure 1).

Strains B74, B75, and B80, were isolated from trachea of birds on farm 2, were resistant to COL, GEN, SUT, and positive for the resistance genes *mcr-1*, *blaTEM*, *tetA*, *tetB*. Variability was observed in the presence of the genes *intl-1*, *pcoD*, *sulI*, *aadA*, *aac3-VIb*, *qacE*Δ. As for plasmids, all were positive for the replicons *B/O* and *FIB*, and only the B80 strain was positive for *I1*. As for the presence of virulence genes, *iroN* and *iutA* were detected in the three strains. Regarding the classification of the phylogenetic group, all three were classified as phylogenetic group F (Figure 1).

Strains B66 and B100 were isolated from the cloaca of birds on farm 2, both showed phenotypic resistance to COL and SUT, with only strain B66 showing resistance to CIP. The B66 strain harbored the *tetA* resistance gene, while the *mcr-1* gene was detected only in B100. As for the detection of plasmid replicons, *IncI2*, *L/M*, *HI2*, *N*, *HII* were detected only in the B100 strain. Virulence genes were not detected in either strain and both strains classified as phylogenetic group B1 (Figure 1).

Evaluating the profile of strains B39 and B42, it was found that both were isolated from the trachea of birds on farm 1 and presented phenotypic resistance to COL, however, the *mcr-1* gene was detected only in B42, while the *tetA* gene was detected in both. The *IncI2* plasmid and *B/O* plasmid replicons were detected in both strains, however, plasmid replicons *FIB* and *II* were present only in B42. As for virulence genes, *iss* and *iutA* were detected only in the B42 strain and both strains classified as phylogenetic group B1 (Figure 1).

## Discussion

When analyzing the phenotypic resistance against the tested antimicrobials, it was found that *E. coli* from all three farms studied showed a high prevalence of colistin resistance (100% farm 1 cloaca, 95% farm 1 trachea, 92% farm 2 cloaca, 95% farm 2 trachea, 87% farm 3 cloaca, 100% farm 3 trachea). When evaluating *E. coli* from a Vietnamese broiler farm, Nguyen et al. (Nguyen et al., 2016) found 22% of isolates were resistant to colistin. Similarly, Fernandes et al. (Fernandes et al., 2016b) who, when evaluating a collection of *E. coli* strains collected from broiler chickens between 2000 and 2016 in Brazil, found that 40% were resistant to colistin. In a recent study carried out in Iran by Azizpour & Saeidi (Azizpour and Saeidi Namin, 2018) it was observed that 68.5% were resistant to colistin. Thus, our data appears to show that the colisitin resistance in poultry continues to persist in Brazil.

High levels of resistance were also found to the antimicrobial SUT on farms 1 (73% cloaca, 76% trachea) and 2 (77% cloaca and 85% trachea), we believe that such high levels may be related the historical context of a high density of breeders and the previous use of this class of antimicrobials in poultry flocks in the mountain region of Rio de Janeiro. However, the values observed on farm 3 (12% cloaca, 25% trachea) were considerably lower when compared to farms 1 and 2, which may be related to the low density of poultry producers and the recent trend toward organic and free-range poultry breeders in the northern region of the state. High levels of resistance to SUT (80%) were also observed by Azizpour & Saeidi (Azizpour and Saeidi Namin, 2018).

*E. coli* strains also showed resistance to gentamicin. Isolates from farm 1 displayed resistance in 31% of cloaca strains and 28% in trachea strains and farm 2, 52% in those that were present in the cloaca and 81% in trachea strains, however, on farm 3, no resistance was observed to this drug. These levels are also similar to those reported by Nguyen and collaborators (Nguyen et al., 2016) where 42% of *E. coli* from broilers were resistant. An additional survey carried out during visits to the farms of the current study found that the growth promoter enramycin was verified as in use on farms 1 and 2 and colistin on farm 3, enramycin is a polypeptide antibiotic. Thus, the resistance verified in phenotypic tests could be explained by the use of enramycin as a growth promoter, as observed by Costa et al. (Costa et al., 2017).

On examination of ciprofloxacin resistance data, the following results were noted 58% and 28% of the strains (cloaca and trachea, respectively) from farm 1 were resistant, on farm 2, 22% of the cloaca strains and 33% of trachea, in addition to 25% of cloaca and 75% of trachea in strains belonging to farm 3. Nguyen and collaborators (Nguyen et al., 2016) found that 73% of 90 isolates examined displayed resistance to ciprofloxacin when studying *E. coli* of avian origin in Vietnam. Abdi-Hachesoo et al. (Abdi-Hachesoo et al., 2017), when researching resistance to quinolones in broiler chicken farms in Iran, found 80% of strains of *E. coli* isolated were resistant from 30-day-old broilers, results similar to those were observed by Azizpour & Saeidi (Azizpour and Saeidi Namin, 2018) who noted a prevalence of 77% in broilers. Ciprofloxacin belongs to the quinolone class, as does enrofloxacin, which is widely used in therapeutic and prophylactic forms in the field. In the survey carried out on the farms during visits, farm 1 and farm 3 had a history of recent use of enrofloxacin. Farm 1 used the antimicrobial in the production batch prior to the survey and on farm 3, the use of the drug occurred in the last three months prior to collection, on both farms (farms 1 and 3) it was used to contain an outbreak of colibacillosis. This antimicrobial has, however, seen considerably limited use in some regions of the world for example in the US where it is no longer approved for use in poultry (FDA, 2005) and this is also the case for the European Union (EU) (EU, 2018).

Considerable levels of resistance to amoxicillin were also observed, with *E. coli* strains from cloacal isolates from farm 1 showing the highest prevalence (57%) followed by trachea strains on farm 1 (38%), cloaca on farm 2 (37%), trachea from farm 2 (28%) and trachea from farm 3 (25%), no resistance was observed in the cloacal strains of farm 3. This resistance may be related to the production of beta-lactamase as it was observed that 52/107 (48%) strains of *E. coli* were suspected of producing this enzyme based on the results of the disk diffusion test.

The most prevalent gene detected in this study was *mcr-1* which was detected in 58% (62/107) of all strains examined, all of which showed phenotypic resistance to colistin, but 39% (40/102) were resistant in the phenotypic test and did not show the presence of any *mcr-*associated gene when examined genotypically, demonstrating the need for future studies regarding the genetic variability of *mcr* and other potential causes of resistance (Yin et al., 2017).

The implementation of IN-45 as of 22 November 2016 in Brazil, prohibits the use of colistin as an additive in feed, is an attempt by the Ministry of Agriculture, Livestock and Supply to reduce the levels of resistance to colistin found in the field. However, it is too early to say whether such a measure will have an effect, since the use of the therapeutic form is currently still approved for use.

Transposons are genetic elements that move in the genome through the action of the enzyme transposase. This movement can occur both within the chromosome and between chromosome and plasmid. Transposons can contain integrons, facilitating the transmission of resistance genes between bacteria. The detection of the *intl1* gene is correlated with the presence of integrons, which are genetic elements that contain a site-specific recombination system capable of integrating, expressing specific DNA elements, called gene cassettes (Hall and Collis, 1995). Integrons consist of three elements: the gene encoding tyrosine recombinase (integrase, encoded by the *intl* gene), required for recombination specific site of the gene cassettes within the integron, the site specific recombination site *attI* and an open reading frame (Gillings, 2014) *qacE*Δ*1* and sulfonamides.

The presence of the gene encoding the transposase enzyme (*iseC12*) was also detected in 13% (14/107) of the *E. coli* strains examined and 10% (6/62) of the positive *mcr-1* strains. The transposons are recognized by integrase; and the promoter (Pc) located upstream of the integration site, is necessary for efficient transcription and expression of the gene cassette present in the integron. Most cassettes present in integrons already described encode resistance determinants, and these genetic elements appear to play an important role in the spread of antimicrobial resistance in Gram negative bacteria (Ploy et al., 2000).

In the strains B75 and B93, which were positive for the *intl1* gene, the concomitant presence of the resistance genes to quaternary ammonia (*qacE*Δ*1*) and sulfonamides (*sulI*) was observed, such characteristics are related to the presence of the class 1 integron, which is conserved in its region downstream the referred genes (Recchia and Hall, 1997).

The high prevalence of APEC-like strains observed on farm 1 could be associated with the occurrence of a collibacilosis case, due to omphalitis, in the first week of life of the animals that made up the batch analyzed in the second collection. The disease was controlled with antimicrobial treatment and disposal of the carcasses of dead animals. Despite these management approaches, it was found that 31.1% (19/61) of cloaca isolates were classified as APEC-like strains (Table 1 and Figure 2C). This data contrasts with another study of *E. coli* cloaca isolates from Brazil (de Oliveira et al., 2015) where 53% of the isolates were classified as APEC-like strains.

When the results of phylogenetic group analysis were compared – most of the isolates in the current study classified as B1 (38%) and A (20%). These results are considerably different from those observed by Rocha et al. (Rocha et al., 2017) who examined APEC and UPEC strains and found that the most common phylogenetic group in APEC was phylogenetic group D (31%) and phylogenetic B2 was most prevalent in the UPEC strains (53%). In the studies by Rocha et al. (Rocha et al., 2017) phylogenetic group B1 was present in only 6% of the UPEC strains. In studies of APEC from Brazil (Barbieri et al., 2015; Braga et al., 2016), they found the majority of their collections classified as phylogenetic groups D with only 7.6% (Barbieri et al., 2015) and 13.3% (Braga et al., 2016) classified as B1. In a study of AFEC (cloacal swabs) from Egypt the most frequently detected phylogenetic groups were A (46.6%) with 33.3% of isolates examined classifying as B1 (Hussein et al., 2013). In a study of retail meat *E. coli* from 2013 from Brazil the most frequent phylogenetic group found was B1 (37.2%) while a 2007 study found phylogenetic group D was most common with a prevalence of 34.5% (Koga et al., 2015).

When PFGE data was assessed, we found significant diversity in the fingerprint profiles of all *E. coli* examined. These data are comparable to other works (Bergeron et al., 2012; Hussein et al., 2013; Barbieri et al., 2015; Braga et al., 2016; de Oliveira et al., 2020) showing that often disease outbreaks are linked to more than one strain of organism. Pulsed field gel electrophoresis is known as a standard tool for pathogen subtyping and has significant application in the identification of outbreak strains, but it is generally found not to be useful for APEC because of the great diversity of strains linked with disease, and in particular the diversity of strains on a single farm that are linked with disease make it difficult to use in tracing the source of the outbreak. Of note in this study, however, strains with the same profile were found between isolates B74, B75, B80 that were isolated from trachea of different birds on farm 2; between B66 and B100 that were isolated from the cloaca of different birds on farm 2; and between B39 and B42 were isolated from trachea of different birds on farm 1 (Figure 1).

## Conclusion

The evaluation of the bacterial microbiota present in samples of cloaca and trachea of broilers found a high prevalence of *E. coli* in both the cloacal sample and tracheal swabs of birds at the various farms.

When assessing antimicrobial resistance in isolated strains, it was noted that the resistance profile varied according to the breeding system and history of antimicrobial use on each farm.

*E. coli* strains were found with phenotypes suggestive of ESBL and AmpC beta-lactamase production.

Phenotypic resistance to colistin was the most prevalent trait among the *E. coli* isolates examined, which was accompanied by a high prevalence of detection of the *mcr-1* gene. Correlations were also observed between the presence of the *mcr-1* gene and the plasmids *HII, IncI2*, *B/O*, and *FIB*. Although it cannot be confirmed that the *mcr-1* gene is located on plasmids, the occurrence of both in the same individual isolate is considered a risk factor, since plasmids can carry the resistance gene and favor its dispersion.

High genetic variability of *E. coli* strains was observed with prevalence of the phylogenetic group B1, related to commensal strains. However, the analysis of the virulence profile detected a high number of APEC-like strains, highlighting the importance of monitoring, cleaning, and disinfecting the environment, control of people and vehicles and the sanitary condition of the sheds between flocks, in order to avoid future infections, occurrence of colibacillosis and consequent economic losses.

## Data Availability Statement

The original contributions presented in the study are included in the article/Supplementary Material, further inquiries can be directed to the corresponding authors.

## Author Contributions

NB design the study, carried out the research, data analysis, and drafting of the manuscript. RP performed sampling collection, analysis of farms and farm visits. DM performed analysis for genes. LN provided assistance in drafting the manuscript and provided supplies for the study. MS provided assistance for sampling collection and provided supplies for the study. CL helped design the study, draft the manuscript, and provided materials for the study. All authors contributed to the article and approved the submitted version.

## Conflict of Interest

The authors declare that the research was conducted in the absence of any commercial or financial relationships that could be construed as a potential conflict of interest.

## Supplementary Material

Supplementary Table 1 Source of isolates examined in this study.

Supplementary Table 2 Primers used in this study.

Supplementary Table 3A Zone Diameter and MIC Breakpoints used on this study.

Supplementary Table 3B Multi Drug Resistance Among Isolates Examined.

Supplementary Table 3C Antimicrobial resistance profiles.

Supplementary Table 4A Chi-square analysis of the association between the presence of two single genes or antimicrobial resistance traits.

Supplementary Table 4B Statistical analysis based on site of isolation.

Supplementary Table 4C Prevalence analysis based on site of isolation.

Supplementary Table 4D All data.
