# Supplementary material for: mcr-1 Identified in Fecal Escherichia coli and Avian Pathogenic E. coli (APEC) From Brazil
Source: Front Microbiol. 2021 Apr 20;12:659613. doi: 10.3389/fmicb.2021.659613 (PMC8093808; doi:10.3389/fmicb.2021.659613)
Supplement: Supplementary Table 2 — Primers used in this study. [file Data_Sheet_2.DOCX]

**Supplement Table 2:**

| Description | Gene | Amplicon size (bp) | Primer Sequence (5'-3') | Reference |
| --- | --- | --- | --- | --- |
| **Antimicrobial associated resistance genes** | | | | |
| colistin resistance | *mcr1* | 1257 | CGGTCAGTCCGTTTGTTC | (1) |
|  |  |  | CTTGGTCGGTCTGTAGGG | (1) |
|  | *mcr2* | 378 | TGTTGCTTGTGCCGATTGGA | (2) |
|  |  |  | AGATGGTATTGTTGGTTGCTG | (2). |
|  | *mcr3* | 814 | TTG GCACTGTATTTTGCATTT- | (3) |
|  |  |  | TTAACGAAATTGGCTGGAACA | (3) |
|  | *mcr4* | 669 | ATTGGGATAGTCGCCTTTTT | (4) |
|  |  |  | TTACAGCCAGAATCATTATCA | (4) |
|  | *mcr5* | 1049 | ATGCGGT TGTCTGCATTTATC | (5) |
|  |  |  | TCATTGTGGTTGTCCTTTTCTG | (5) |
|  | *mcr6* | 556 | GTCCGGTCAATCCCTATCTGT | (6) |
|  |  |  | ATCACGGGATTGACATAGCTAC | (6) |
|  | *mcr7* | 892 | TGCTCAAGCCCTTCTTTTCGT | (6) |
|  |  |  | TTCATCTGCGCCACCTCGT | (6) |
|  | *mcr8* | 667 | AACCGCCAGAGCACAGAATT | (6) |
|  |  |  | TTCCCCCAGCGATTCTCCAT | (6) |
|  | *mcr9* | 1599 | GGT AGT TAT TCC GCT GG | (7) |
|  |  |  | TCG CGG TCA GGA TTA AC | (7) |
| silver resistance | *silP* | 603 | ACACCCCGGCCTGGGCTCCTT |  |
|  |  |  | TGCGGGCACGGGAACAAACCTC |  |
| integrase | *intI1* | 545 | CACTCCGGCACCGCCAACTTTC |  |
|  |  |  | GAACGGGCATGCGGATCAGTGAG |  |
| cooper resistance | *pcoD* | 502 | GGCGCCCAGAATGATAATCGCAACA |  |
|  |  |  | GGGCGTGGCGCTGGCTACACTT |  |
| sulfa resistance | *sulI* | 462 | CGCCGCTCTTAGACGCCCTGTCC |  |
|  |  |  | CAACGGTGGCGCCCAAGAAGGAT |  |
| transposase | *ISEc12* | 404 | CGCGGCCACGTAAACCGAAAGATAAA |  |
|  |  |  | GCGCGGGTGCACAGCAACCTC |  |
| aminoglycoside resistance | *aadA* | 365 | TAACGGCGCAGTGGCGGTTTTCA |  |
|  |  |  | AAGCTCGCCGCGTTGTTTCATCAAG |  |
| gentamicin resistance | *aac3-VI* | 302 | GGGCAAGCGCCGCGTCACTTATT |  |
|  |  |  | CGCGGCGTTGTTTCGGCTTCA |  |
| quarternary amonium resistance | *qac delta1* | 246 | TCGGCCTCCGCAGCGACTTCC |  |
|  |  |  | CTTGCCCCTTCCGCCGTTGTCTAAT |  |
| ampicillin resistance | *blaTEM* | 558 | ATGTGCGCGGAACCCCTATTTGTTTA |  |
|  |  |  | AAAAAGCGGTTAGCTCCTTCGGTCCT |  |
| gentamicin resistance | *aac3-VI* | 502 | GGCACCCGCGACGCCCTGGTCCAAAAG |  |
|  |  |  | GGGCCCGGCGCCGATCGACAGGATTT |  |
| tetracycline resistance | *tetB* | 446 | AACGCGTGAAGTGGTTCGGTTGGT |  |
|  |  |  | TTCGCCCCATTTAGTGGCTATTCTTC |  |
| tetracycline resistance | *tetA* | 372 | CGGGGCGACTGGGGCGGTAGC |  |
|  |  |  | CAAAGCGCGGCCGGCACCTGT |  |
| chaperone | *groEL* | 318 | CGCCGGCATGAACCCGATGGACCTCA |  |
|  |  |  | TCGGCCTGCATCGACTGCGGGTTGTTG |  |
| gentamicin resistance | *aph(3)IA* | 278 | TCGGGCAATCAGGTGCGACAATCTA |  |
|  |  |  | TGCCAGCGCATCAACAATATTTTCACC |  |
| trimethoprim resistance | *dfr17* | 243 | ATATCCCGTGGTCAGTAAAAGGTG |  |
|  |  |  | GACCCCCGCCAGAGACATA |  |
| **APEC minimal predictors** | | | | |
| Salmochelin siderophore receptor gene | *iroN* | 553 | AATCCGGCAAAGAGACGAACCGCCTGTA | (8) |
|  |  |  | GTTCGGGCAACCCCTGCTTTGACTTTGA | (8) |
| Episomal outer membrane protease gene | *ompT* | 496 | TCATCCCGGAAGCCTCCCTCACTACTAT | (8) |
|  |  |  | TAGCGTTTGCTGCACTGGCTTCTGATAC | (8) |
| Putative avian hemolysin F | *hlyF* | 450 | GGCCACAGTCGTTTAGGGTGCTTACC | (8) |
|  |  |  | GGCGGTTTAGGCATTCCGATACTCAG | (8) |
| Episomal increased serum survival gene | *iss* | 323 | CAGCAACCCGAACCACTTGATG | (8) |
|  |  |  | AGCATTGCCAGAGCGGCAGAA | (8) |
| Aerobactin siderophore receptor gene | *iutA* | 302 | GGCTGGACATCATGGGAACTGG | (8) |
|  |  |  | CGTCGGGAACGGGTAGAATCG | (8) |
| ***Plasmid Replicon genes*** | | | | |
| plasmid replicon typing | *incI2* | 276 | AGTCACAGTATCGCGCCTTT | (9) |
|  |  |  | TTACGAGCCGAGTGAACAGA | (9) |
| plasmid replicon typing | *T* | 750 | TTGGCCTGTTTGTGCCTAAACCAT | (10) |
|  |  |  | CGTTGATTACACTTAGCTTTGGAC | (10) |
| plasmid replicon typing | *P* | 534 | CTATGGCCCTGCAAACGCGCCAGAAA | (10) |
|  |  |  | TCACGCGCCAGGGCGCAGCC | (10) |
| plasmid replicon typing | *A/C* | 465 | GAGAACCAAAGACAAAGACCTGGA | (10) |
|  |  |  | ACGACAAACCTGAATTGCCTCCTT | (10) |
| plasmid replicon typing | *FIC* | 262 | GTGAACTGGCAGATGAGGAAGG | (10) |
|  |  |  | TTCTCCTCGTCGCCAAACTAGAT | (10) |
| plasmid replicon typing | *B/O* | 159 | GCGGTCCGGAAAGCCAGAAAAC | (10) |
|  |  |  | TCTGCGTTCCGCCAAGTTCGA | (10) |
| plasmid replicon typing | *Y* | 765 | AATTCAAACAACACTGTGCAGCCTG | (10) |
|  |  |  | GCGAGAATGGACGATTACAAAACTTT | (10) |
| plasmid replicon typing | *FIB* | 702 | GGAGTTCTGACACACGATTTTCTG | (10) |
|  |  |  | CTCCCGTCGCTTCAGGGCATT | (10) |
| plasmid replicon typing | *FIA* | 462 | CCATGCTGGTTCTAGAGAAGGTG | (10) |
|  |  |  | GTATATCCTTACTGGCTTCCGCAG | (10) |
| plasmid replicon typing | *FIIA* | *270* | CTGTCGTAAGCTGATGGC | (10) |
|  |  |  | CTCTGCCACAAACTTCAGC | (10) |
| plasmid replicon typing | *W* | *242* | CCTAAGAACAACAAAGCCCCCG | (10) |
|  |  |  | GGTGCGCGGCATAGAACCGT | (10) |
| plasmid replicon typing | *K/B* | 160 | GCGGTCCGGAAAGCCAGAAAAC | (10) |
|  |  |  | TCTTTCACGAGCCCGCCAAA | (10) |
| plasmid replicon typing | *L/M* | 785 | GGATGAAAACTATCAGCATCTGAAG | (10) |
|  |  |  | CTGCAGGGGCGATTCTTTAGG | (10) |
| plasmid replicon typing | *Hl2* | *644* | TTTCTCCTGAGTCACCTGTTAACAC | (10) |
|  |  |  | GGCTCACTACCGTTGTCATCCT | (10) |
| plasmid replicon typing | *N* | 559 | GTCTAACGAGCTTACCGAAG | (10) |
|  |  |  | GTTTCAACTCTGCCAAGTTC | (10) |
| plasmid replicon typing | *HII* | 471 | GGAGCGATGGATTACTTCAGTAC | (10) |
|  |  |  | TGCCGTTTCACCTCGTGAGTA | (10) |
| plasmid replicon typing | *X* | 376 | AACCTTAGAGGCTATTTAAGTTGCTGAT | (10) |
|  |  |  | TGAGAGTCAATTTTTATCTCATGTTTTAGC | (10) |
| plasmid replicon typing | *II* | 139 | CGAAAGCCGGACGGCAGAA | (10) |
|  |  |  | TCGTCGTTCCGCCAAGTTCGT | (10) |
| **Phylogenetic Typing** | | | | |
| Quadruplex PCR | *chuA* | 288 | ATGGTACCGGACGAACCAAC | (11) |
|  |  |  | TGCCGCCAGTACCAAAGACA | (11) |
| Quadruplex PCR | *yjaA* | 211 | CAAACGTGAAGTGTCAGGAG | (12) |
|  |  |  | AATGCGTTCCTCAACCTGTG | (12) |
| Quadruplex PCR | TspE4.C2 | 152 | CACTATTCGTAAGGTCATCC | (12) |
|  |  |  | AGTTTATCGCTGCGGGTCGC | (12) |
| Quadruplex PCR | *arpA* | 400 | AACGCTATTCGCCAGCTTGC | (12) |
|  |  |  | TCTCCCCATACCGTACGCTA | (12) |
| Group E | *arpA** | 301 | GATTCCATCTTGTCAAAATATGCC | (13) |
|  |  |  | GAAAAGAAAAAGAATTCCCAAGAG | (13) |
| Group C | *trpA* | 219 | AGTTTTATGCCCAGTGCGAG | (13) |
|  |  |  | TCTGCGCCGGTCACGCCC | (13) |

**References:**

1. Liu YY, Wang Y, Walsh TR, Yi LX, Zhang R, Spencer J, et al. Emergence of plasmid-mediated colistin resistance mechanism MCR-1 in animals and human beings in China: a microbiological and molecular biological study. *Lancet Infect Dis* (2016) 16(2):161-8. doi: 10.1016/S1473-3099(15)00424-7. PubMed PMID: 26603172.

2. Xavier BB, Lammens C, Ruhal R, Kumar-Singh S, Butaye P, Goossens H, et al. Identification of a novel plasmid-mediated colistin-resistance gene, *mcr-2*, in *Escherichia coli*, Belgium, June 2016. *Euro Surveill* (2016) 21(27). Epub 2016/07/16. doi: 10.2807/1560-7917.Es.2016.21.27.30280. PubMed PMID: 27416987.

3. Yin W, Li H, Shen Y, Liu Z, Wang S, Shen Z, et al. Novel Plasmid-Mediated Colistin Resistance Gene *mcr-3* in *Escherichia coli*. *MBio* (2017) 8(3). Epub 2017/06/29. doi: 10.1128/mBio.00543-17. PubMed PMID: 28655818; PubMed Central PMCID: PMCPMC5487729.

4. Carattoli A, Villa L, Feudi C, Curcio L, Orsini S, Luppi A, et al. Novel plasmid-mediated colistin resistance *mcr-4* gene in *Salmonella* and *Escherichia coli,* Italy 2013, Spain and Belgium, 2015 to 2016. *Euro Surveill* (2017) 22(31). Epub 2017/08/12. doi: 10.2807/1560-7917.Es.2017.22.31.30589. PubMed PMID: 28797329; PubMed Central PMCID: PMCPMC5553062.

5. Borowiak M, Fischer J, Hammerl JA, Hendriksen RS, Szabo I, Malorny B. Identification of a novel transposon-associated phosphoethanolamine transferase gene, *mcr-5*, conferring colistin resistance in d-tartrate fermenting *Salmonella enterica* subsp. enterica serovar Paratyphi B. *J Antimicrob Chemother* (2017) 72(12):3317-24. Epub 2017/09/30. doi: 10.1093/jac/dkx327. PubMed PMID: 28962028.

6. Yang F, Shen C, Zheng X, Liu Y, El-Sayed Ahmed MAE, Zhao Z, et al. Plasmid-mediated colistin resistance gene *mcr-1* in *Escherichia coli* and *Klebsiella pneumoniae* isolated from market retail fruits in Guangzhou, China. *Infect Drug Resist* (2019) 12:385-9. Epub 2019/02/28. doi: 10.2147/idr.S194635. PubMed PMID: 30809099; PubMed Central PMCID: PMCPMC6377047.

7. Kieffer N, Royer G, Decousser JW, Bourrel AS, Palmieri M, Ortiz De La Rosa JM, et al. *mcr-9*, an Inducible Gene Encoding an Acquired Phosphoethanolamine Transferase in *Escherichia coli,* and Its Origin. *Antimicrob Agents Chemother* (2019) 63(9). Epub 2019/06/19. doi: 10.1128/aac.00965-19. PubMed PMID: 31209009; PubMed Central PMCID: PMCPMC6709461.

8. Johnson TJ, Wannemuehler Y, Doetkott C, Johnson SJ, Rosenberger SC, Nolan LK. Identification of Minimal Predictors of Avian Pathogenic *Escherichia coli* Virulence for Use as a Rapid Diagnostic Tool. *Journal of Clinical Microbiology* (2008) 46(12):3987-96. doi: 10.1128/jcm.00816-08. PubMed PMID: WOS:000261247900019.

9. Zhao F, Zong Z. *Kluyvera ascorbata* Strain from Hospital Sewage Carrying the *mcr-1* Colistin Resistance Gene. *Antimicrob Agents Chemother* (2016) 60(12):7498-501. Epub 2016/09/28. doi: 10.1128/aac.01165-16. PubMed PMID: 27671069; PubMed Central PMCID: PMCPMC5119035.

10. Johnson TJ, Wannemuehler YM, Johnson SJ, Logue CM, White DG, Doetkott C, et al. Plasmid replicon typing of commensal and pathogenic Escherichia coli isolates. *Applied and Environmental Microbiology* (2007) 73(6):1976-83. doi: 10.1128/aem.02171-06. PubMed PMID: WOS:000245156800035.

11. Clermont O, Bonacorsi S, Bingen E. Rapid and simple determination of the Escherichia coli phylogenetic group. *Applied and Environmental Microbiology* (2000) 66(10):4555-8. doi: 10.1128/aem.66.10.4555-4558.2000. PubMed PMID: WOS:000089649700061.

12. Clermont O, Christenson JK, Denamur E, Gordon DM. The Clermont *Escherichia coli* phylo-typing method revisited: improvement of specificity and detection of new phylo-groups. *Environmental Microbiology Reports* (2013) 5(1):58-65. doi: 10.1111/1758-2229.12019. PubMed PMID: WOS:000314474500005.

13. Lescat M, Clermont O, Woerther PL, Glodt J, Dion S, Skurnik D, et al. Commensal Escherichia coli strains in Guiana reveal a high genetic diversity with host-dependant population structure. *Environmental Microbiology Reports* (2013) 5(1):49-57. doi: <https://doi.org/10.1111/j.1758-2229.2012.00374.x>.
